# Corrigendum: Down-Regulated FOXO1 in Refractory/Relapse Childhood B-Cell Acute Lymphoblastic Leukemia

**DOI:** 10.3389/fonc.2020.629875

**Published:** 2020-12-09

**Authors:** Qingqing Zheng, Chuang Jiang, Haiyan Liu, Wenge Hao, Pengfei Wang, Haiying Huang, Ziping Li, Jiabi Qian, Maoxiang Qian, Hui Zhang

**Affiliations:** ^1^Department of Hematology/Oncology, Guangzhou Women and Children’s Medical Center, Guangzhou, China; ^2^Shanghai Children’s Medical Center, School of Medicine, Shanghai Jiao Tong University, Shanghai, China; ^3^Institute of Pediatrics and Department of Hematology and Oncology, Children’s Hospital of Fudan University, the Shanghai Key Laboratory of Medical Epigenetics, International Co-laboratory of Medical Epigenetics and Metabolism, Ministry of Science and Technology, Institutes of Biomedical Sciences, Fudan University, Shanghai, China

**Keywords:** acute lymphoblast leukemia, risk stratification, relapse, *FOXO1*, MRD—minimal residual disease

In the published article, there was an error in affiliation 3. Instead of “Shanghai Key Laboratory of Medical Epigenetics, International Co-Laboratory of Medical Epigenetics and Metabolism, Department of Hematology and Oncology, Institute of Pediatrics, Institutes of Biomedical Sciences, Children’s Hospital of Fudan University, Ministry of Science and Technology, Fudan University, Shanghai, China”, it should be “Institute of Pediatrics and Department of Hematology and Oncology, Children’s Hospital of Fudan University, the Shanghai Key Laboratory of Medical Epigenetics, International Co-laboratory of Medical Epigenetics and Metabolism, Ministry of Science and Technology, Institutes of Biomedical Sciences, Fudan University, Shanghai, China”.

The authors apologize for this error and state that this does not change the scientific conclusions of the article in any way. The original article has been updated.

